# Active and Healthy Aging After COVID-19 Pandemic in Portugal and Other European Countries: Time to Rethink Strategies and Foster Action

**DOI:** 10.3389/fpubh.2021.700279

**Published:** 2021-07-02

**Authors:** Andreia Costa, Gisele Câmara, Miguel Telo de Arriaga, Paulo Nogueira, José Pereira Miguel

**Affiliations:** ^1^Instituto de Saúde Ambiental (ISAMB), Faculdade de Medicina, Universidade de Lisboa, Lisboa, Portugal; ^2^Nursing Research, Innovation and Development Centre of Lisbon (CIDNUR), Nursing School of Lisbon, Lisbon, Portugal; ^3^Direção-Geral da Saúde, Ministério da Saúde, Lisboa, Portugal; ^4^CRC-W – Católica Research Centre for Psychological, Family and Social Wellbeing, Faculdade de Ciências Humanas, Universidade Católica Portuguesa, Lisboa, Portugal; ^5^Laboratório de Biomatemática, Faculdade de Medicina, Universidade de Lisboa, Lisboa, Portugal

**Keywords:** active and healthy aging, Portugal, public health, policy and institutional actions, COVID-19, Europe

## Abstract

The population aging in Europe imposes challenges to societies that require adaptations and responses at various levels to minimize impacts and figuring out opportunities. Portugal has been committed to the World Health Organization and European Union's values and policy frameworks concerning active and healthy aging. In 2017, an inter-ministerial working group developed the National Strategy for Active and Healthy Aging. In the face of the COVID-19 pandemic that exposed the vulnerabilities of older populations, the launch of the Decade of Healthy Aging 2021–2030 and its baseline report and the 2018 Active Aging Index Analytical Report may constitute an opportunity to strategically think about the aging of the population as a national purpose in Portugal and in the other European countries that face similar challenges.

## Introduction

Life expectancy at birth has increased by about 10 years over the last five decades in Europe (1). The increase in life expectancy, combined with the decline in the birth rate in the previous century, has led to a reversal of the age pyramid in many European countries (2).

Portugal is the 4th country in the 28 European Union (EU) with the highest percentage of older people, behind Italy, the most aged country in EU, Germany and Greece (2, 3). In Portugal, a baby born in 2018 could expect to live 78 years if a boy and 83.5 if a girl (4). Between 1970 and 2019, the percentage of the population aged 65 or more in Portugal increased from 9.7 to 22%, and the portion of the population aged 14 years old or less decreased almost by half, from 28.5 to 13.6% (5).

Despite the similarities between southern European countries, especially Spain, Italy, and Greece, in terms of percentages of the elderly population and average life expectancy, healthy life expectancy at age 65 in Portugal (7.3 years) remains 3 years below the average of the current 27 countries in European Union (10.3 years), 3.1 years below Italian (10.4 years) and 5.1 years below Spanish (12.4 years), being similar only to Greek (7.9 years) (6).

The burden of disease and the reduction of well-being affect the elderly people, their families, and health, social and economic systems (7). Older people with health issues, disabilities or lack of autonomy need more health care and social support from their families, social economy institutions and health services (8). This is particularly serious considering that 20% of the Portuguese population aged 65 or over were at risk of poverty or social exclusion in 2019 (9).

These factors impose a substantial impact on society. They require adaptations and responses at various levels, mainly through the support systems, such as health, social security, education, justice, and transportation systems (3, 10). The impact of an aging population on society depends, in part, on the nature of policies that will respond to this new reality (8, 11).

This policy statement seeks to briefly describe the policy options around aging adopted by the European Union and Portugal in recent years and demonstrate the imperative need to act after the COVID-19 pandemic's exposition of older people's vulnerabilities.

## Policy Options and Implications

### Active and Healthy Aging

There is a lot to be done to improve the quality of the years we have been gaining. Active and healthy aging throughout the life cycle has been considered a response to the challenges related to the longevity and aging of the population (10, 12–14).

Active aging was first defined by World Health Organization (WHO) as optimizing health, participation, and safety opportunities to improve quality of life as people age. The term “active” refers to the continuous participation in social, economic, cultural, spiritual, and civic life, meaning it goes far beyond the possibility of being physically and professionally active (10).

European Commission considers active aging as the policy directed toward “helping people stay in charge of their own lives for as long as possible as they age and, where possible, to contribute to the economy and society” (3).

Besides the health conditions, these concepts involve environmental and personal factors such as economic, social and cultural determinants, the physical environment, the health system, gender and other determinants (10, 15).

More recently, WHO adopted the most straightforward term and concept of “healthy aging” referring to the development and maintenance of functional capacity, which contributes to the well-being of elderly people. According to this new conceptual framework, functional capacity results from the interaction of intrinsic capacities (physical capacities and person's mental health) with the environment. The main objective is well-being, a holistic concept that includes all the elements and components of life valued by the person. Thus, more than the result of success and individual motivation, healthy aging reflects the support and opportunities guaranteed by society to maintain the functionality of the elderly people and allow them to experience what they value (13).

All the above conceptualizations highlight the need to develop multidisciplinary and integrated work in active aging promotion. The elderly people must participate in economic, political, social, and cultural life. They should have opportunities to work when they wish and continue to access education programs and training. The potential of older people (capacity, experience and wisdom) should be seen as a solid foundation for future development, enabling society to benefit from it (15).

### Active Aging Index

The Active Aging Index (AAI) is a project managed jointly by the European Commission's Directorate-General for Employment, Social Affairs and Inclusion and the Population Unit of the United Nations Economic Commission for Europe. AAI is a tool to measure the untapped potential of older people for active and healthy aging at national and subnational levels (16).

The AAI's conceptual framework follows a multidimensional perspective, considering the different forms through which older persons contribute to society and the economy: through paid or voluntary work, informal care, political participation, or by keeping healthy, informed, and independent lifestyles even at an advanced age. It also considers environmental factors which enable them to be more active, such as the educational and care systems or the different infrastructures promoting well-being, social cohesion, and digitalization (17).

The 22 indicators grouped in 4 domains used to monitor progress on AAI since 2008 suggest the need for more investment on active aging in Portugal, which scores below the Blue Cluster average of which it is part, together with the Czech Republic, Estonia, Germany, Ireland, Latvia and Lithuania. These countries report low scores in all domains except in Employment, with a problematic situation especially concerning Social Participation, as shown in the 2018 Active Aging Index Analytical Report (17) and summarized in Table 1.

**Table 1:** Trends of the Portuguese, Blue Cluster and 28 European Union's scores for Active Aging Index and its domains between 2008 and 2016.

**Year**	**2008**			**2016**		
**Domains**	**Scores Portugal**	**Scores blue cluster**	**EU (18) average**	**Scores Portugal**	**Scores blue cluster**	**EU (28) average**
Capacity and enabling environment for active aging	47.2	49.7	-	54.2	55.9	57.5
Participation in society	10.1	13.2	-	11.9	15.1	17.9
Independent. healthy and secure living	67.1	66.4	-	67.7	69.8	71.8
Employment	36.6	33.3	-	33.4	37.5	31.1
Global active aging	32.5	32.9	32.2	33.5	36.6	35.8

*Source: European Commission (16)*.

### Portuguese Aging Policies Alignment With WHO and EU

The Constitution of the Portuguese Republic recognizes the right of the dignity of the human being. A particular reference is made to the elderly population regarding economic security and living conditions that respect their personal autonomy and avoid isolation and social marginalization (19). The respect for human dignity is also a principle of the Portuguese Fundamental Health Law (20) and a duty repeatedly present in health professionals' deontological codes (21, 22).

Portugal has been committed to the WHO and European Union's values and strategic objectives concerning active and healthy aging since the Political Declaration and Madrid International Plan of action on aging (15).

In 2004, through the Directorate-General for Health (DGS), the Portuguese Ministry of Health launched the National Program for the Health of the Older People (14). In 2014, the report “Greater Age in Numbers” was created to monitor the over 65 aged population's health (14, 23).

Aligned with The Global Strategy and Action Plan on Aging and Health adopted by the World Health Assembly in May 2016 (24), with the new framework for action on aging and health presented in the World Report on Aging and Health (13), and with the European Union's policies and practices for active and healthy aging (1, 2, 16, 25) the Portuguese Government nominated an inter-ministerial working group to develop the National Strategy for Active and Healthy Aging—ENEAS (26). The strategic plan developed and proposed in 2017 intended to build a society for all ages, where all persons would experience an active, dignified, and healthy aging. To achieve this goal, the working group proposed a set of actions organized into seven groups: Promotion of healthy lifestyles and health surveillance; Comorbidity's management; Training and education throughout the life cycle; Creation of environments that enable integration and participation; Creation of physical environments that ensure safety; and Identification, signaling and support in situation of vulnerability; Implementation, monitoring and research (26).

### COVID-19 Pandemic: Exposing Vulnerabilities of Older People and Offering Clues to Responses_

COVID-19 pandemic exposed dysfunction and fragility in many systems. Still, it also revealed resilience and creativity to save and improve lives and the value of the ecosystems on which we all depend (27).

The pandemic disproportionately affected older people, constituting a higher risk group of developing severe illness worldwide (28). In Portugal, it was the most affected group in terms of mortality (18), need for hospitalization (29) and intensive care (30) and poor quality of life associated with the pandemic combat responses (31).

But age was not a singular risk factor for the elderly. Older people's health status before the pandemic determined their susceptibility to severe illness, their recovery and their longer-term health and well-being. Other disparities have emerged, including ethnicity, gender, income and some living arrangements, such as long-term care facilities, isolation and crowded living situations. It seems that pandemic reinforced the importance of concerted, sustained focus, investment and action to foster healthy aging (27).

It is crucial to analyze carefully the clues offered by the studies designed to better understand social and health pandemic impacts and even apparent paradoxes (28, 32–35) and the responses to the pandemic, from clinical and health services organization issues (32, 36) to policies and international collaboration, as is the case of the quick development of vaccines (37).

Many lessons emerge from the extensive list of publications around the theme, being the importance of Open Access Science one of them (38). The need to adapt health systems, for example, has demonstrated the potential of some promising pathways: eHealth, quarantine management and health and social care more integrated management of COVID-19 patients and suspected cases evolved (32). The different-than-expected effects of some rules imposed on the elderly is another important lesson learned. Prolonged shielding, for example, protected the elderly from being infected by the virus, but restrictions on movement and socialization also resulted in isolation (27).

### The COVID-19 Pandemic, 2018 Active Aging Index Analytical Report and The Decade of Healthy Aging: Implications and Time for Actions

Coinciding with the COVID-19 pandemic, the end of the first strategic period of the Global Strategy and Action Plan on Aging and Health 2016–2020 was the moment for debate and reflection on the launch of the Decade of Healthy Aging 2021–2030 (39) and its baseline report (40). This action plan is seen as an opportunity to align global, national and local policies, with older people, for older people (39).

Four accurate interconnected areas for action are addressed to improve functional ability until 2030: Change how we think, feel and act toward age and aging; Ensure that communities foster the skills of older people; Deliver person-centered integrated care and primary health services responsive to older people; and Provide access to long-term care for older people who need it. The working group also identifies what they call “enablers” to support action: meaningful engagement with older people, families, caregivers and others; building capacity for integrated action across sectors; linking stakeholders to share experience and learn from others; and strengthening data, research and innovation to accelerate implementation (31).

Besides a renewed, multisectoral action framework, built on the evidence that emerged from the 2016–2020 period, the Sustainable Development Goals and the new reality presented by the COVID-19 pandemic, the Decade of Healthy Aging 2021–2030 show a framework for tracking progress (31).

In the same way, the 2018 Active Aging Index Analytical Report provides a range of examples on how the AAI can be used by policymakers, researchers, and other interested parties to identify areas where policies can realize the active potential of older people (27).

According to European Commission, the COVID-19 pandemic exposed the vulnerabilities of an aging population but is not thought likely to have changed this overall positive trend on life expectancy (1).

There is no doubt that it is time for action. These two frameworks may fit perfectly into the need to analyze the current situation in the face of the new reality imposed by the COVID 19 pandemic, rethinking strategies and monitoring the advances.

## Conclusions

In the face of the new challenges imposed by the COVID-19 pandemic, the launch of the 2018 Active Aging Index Analytical Report and the Decade of Healthy Aging 2021–2030 may constitute an opportunity to strategically think about the aging of the population as a national purpose in Portugal and more broadly in Europe.

As WHO, we recognize that what is measured drives action. Adapting ENEAS to the latest policy and monitoring frameworks developed by the European Commission and WHO will allow updating challenges, responses and indicators and obtaining reliable data comparable at national and international levels.

## Author Contributions

AC, GC, MA, PN, and JM contributed to the conception and design of the article. AC, GC, and JM wrote the first draft of the manuscript. PN and MA revised and add new statements and data to the manuscript. All authors contributed to manuscript revision, read, and approved the submitted version.

## Conflict of Interest

The authors declare that the research was conducted in the absence of any commercial or financial relationships that could be construed as a potential conflict of interest.
